# Supplementary material for: A Structural In Silico Analysis of the Immunogenicity of L-Asparaginase from Penicillium cerradense
Source: Int J Mol Sci. 2024 Apr 27;25(9):4788. doi: 10.3390/ijms25094788 (PMC11084778; doi:10.3390/ijms25094788)
Supplement: Supplementary file 1 [file ijms-25-04788-s001.zip › Supplementary/Supplementary Figures .docx]

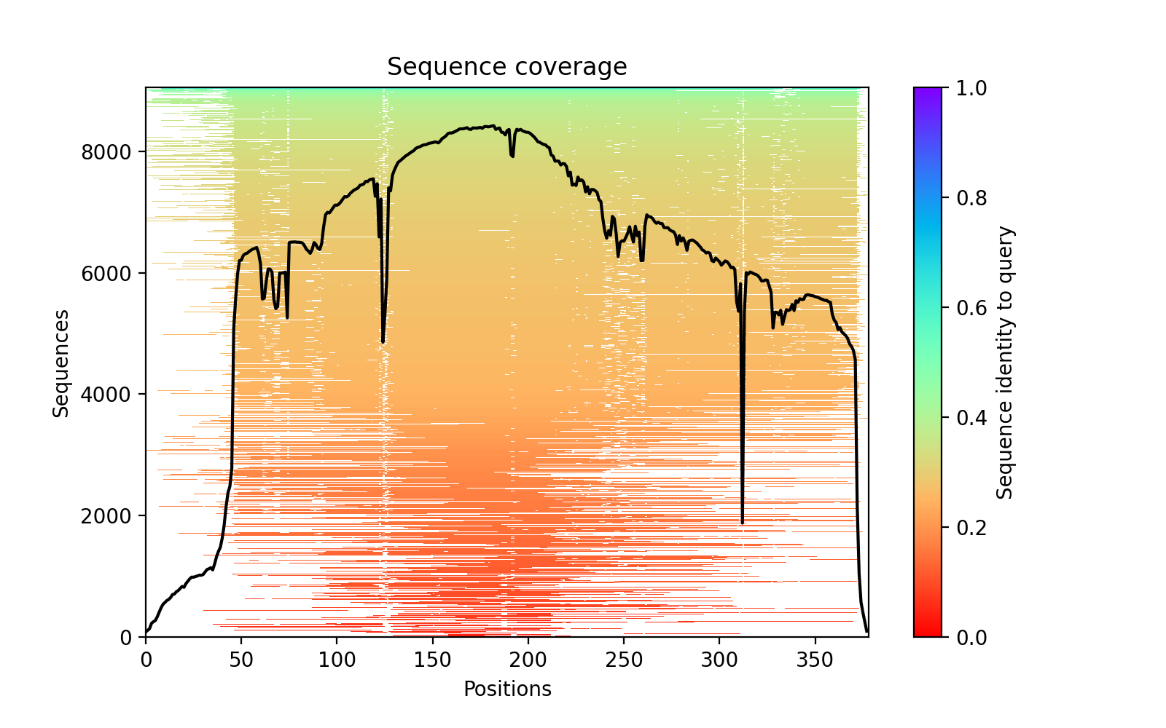


Supplementary Figure S1: Reliability profile of the prediction of the three-dimensional structure of L-asparaginase from P. cerradense by color coding based on internal measurements with AlphaFold2®.


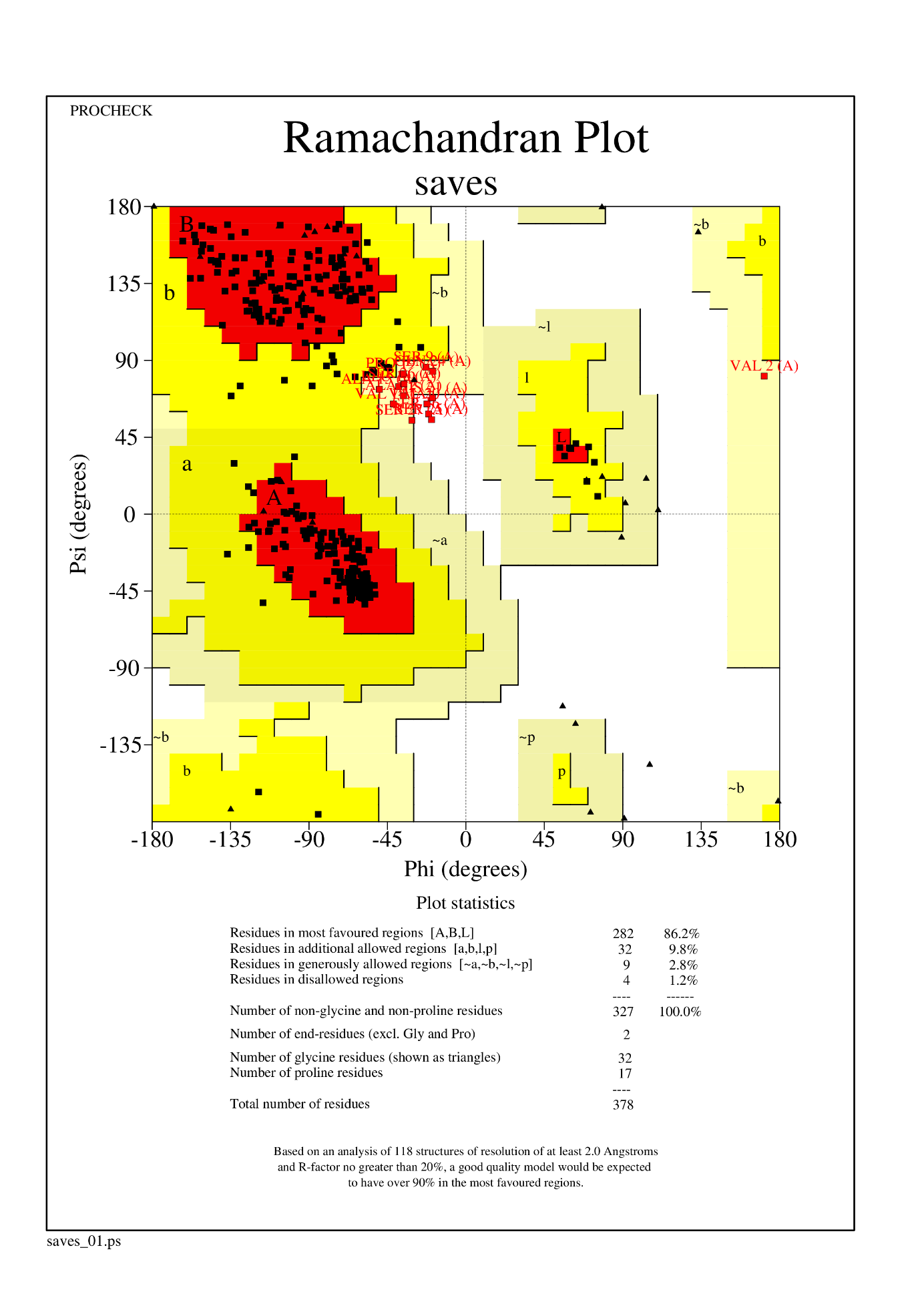


**Supplementary Figure S2** - Ramachandran plot of the structure of L-asparaginase from *P. cerradense* predicted with Alphafold2® representing 96.0% of residues present in the favorable allowed region (red, bright and light yellow).


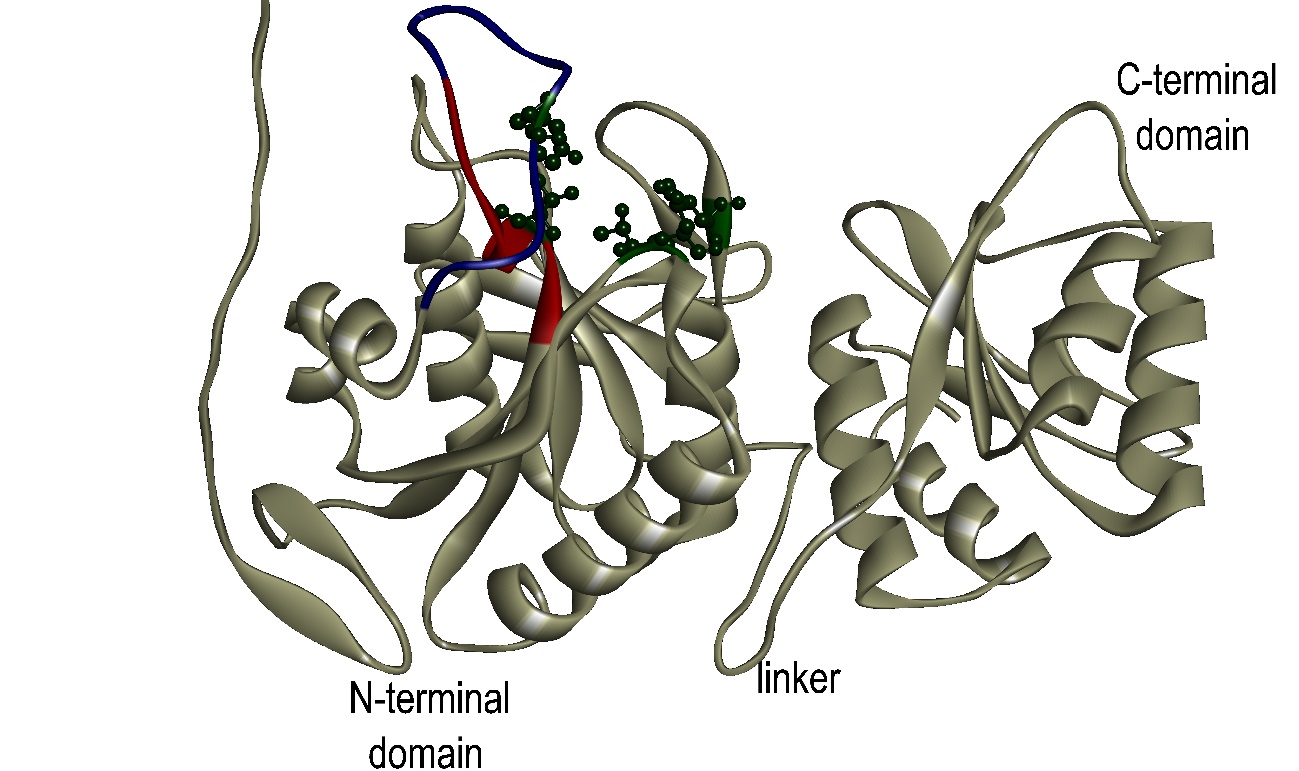


**Supplementary Figure S3:** Monomeric ASNase from *P. cerradense* with the indication of the N-terminal domain, the C-terminal domain, the linker, the HR region in red, the ASFL element in blue, and the conserved residues of the active site in green.
