# Supplementary material for: A Structural In Silico Analysis of the Immunogenicity of L-Asparaginase from Penicillium cerradense
Source: Int J Mol Sci. 2024 Apr 27;25(9):4788. doi: 10.3390/ijms25094788 (PMC11084778; doi:10.3390/ijms25094788)
Supplement: Supplementary file 1 [file ijms-25-04788-s001.zip › Supplementary/Supplementary List S1 - Sequence genes.docx]

**Supplementary List S1** - L-asparaginase gene sequences

> UDP03915.1 L-asparaginase *Penicillium cerradense*

MVIRTIIISLLAIVASGSPVHKPQSSSTKYVYTNSNGLNFTQMNPGLPNITIFGTGGTIAGSGSSSTATTGYTAGAVGILDLIDAVPSMLNVSNIAGVQVANVGSEDITSDILISLSKSINKLVCDDSTMAGAVITHGTDTLEETAFFLDATINCGKPVVIVGAMRPSTATSADGPFNLLEAVTVAASPKAVNRGAMVVMNDRIASAYYVTKTNANTMDTFKAIEMGFLGEMISNTPFFFYPPVKPTGKVEFDITATKEIPRVDILYAYEDMHNDTLYSAVENGAQGIVIAGAGAGGVSTSFNHAIEDVINRFKIPVVQSMRTVNGEVPLSDVNSTSAIHIASGYLNPQKSRILLGLLLSEARTLTDIRSVFSLGTVS

>XP_002563013 L-asparaginase *Penicillium chrysogenum*

MGFSLQALTVSALAITSYASPLIHSRATNTSYTNSNGLTFNHFNGSLPNVTILATGGTIAGTSDDKTATAGYESGALGINTLLSKIPEIFNVANIAAVQAHNVNSGDISSSLLLNLTHTLQATVCDDPTMSGAVITHGTDTLEESAFFIDATVNCGKPIVFVGSMRPATAISADGPMNLVQGVTVAADNDSRDRGALVVLNDRIVSALFATKTHANTMDTFKAYEQGNLGFIVSNKPYFYYPAVQANVKHVVDVSNVDSVPRVDILYAYEDMQVDSLYSAMKNGAKGIVVAGEGAGGVATDFGSAINDIVKKHNIPVVLSHRTVNGEVPTADFTGEDAQTKIASGLFNPQQARILLGLLLAEKKGLKEIREVFLKATVA

>XP_014538187.1 L-asparaginase *Penicillium digitatum*

MGITIQTLAISALVTTSYASPLIYPRAVNTSYTNSNGLTFSHFNGSLPNVTILATGGTIAGTSDDKTTTAGYKSGALGINTLLSGIPDIFKIANIAAIQAHNVNSGDISSSLLLNLTQTLQTQVCDDPTMSGAVITHGTDTLEESAFFLDATVNCGKPIVFVGSMRPSTAISADGPMNLLQGVTVAADKNSRDRGALVVLNDRIVSALFATKTSANTVDTFKAYEQGSLGFIVSNKPYFYYPAVQANAKHVADVSNVDAMPRVDILYAYEDMQIDSIYSAVKNGAKGIVIAGEGAGGVSTDFASAINDIGVKHSIPVVLSHRTVNGEVPTADFTGENAETKIASGMFNPQQSRILLGLLLAEKKGFKEIREVFSKATVA

> KXG45967.1 L-asparaginase *Penicillium griseofulvum*

MGFSLRTLAVSALAITTYASPLISPRTANTSYTNSNGLTFNHFNNSLPNVTILATGGTIAGTSKDKTATAGYKSGALGINTLLSEIPDIFNVANIAAIQANNVNSGDISSSLLLNLTHRLQTEVCDDPTMSGAVITHGTDTLEESAFFIDATVNCGKPIVFVGSMRPSTAISADGPMNLLQGVNVAADKNAKDRGALVVLNDRIVSAFFATKTHANTMDTFQAYEQGSLGFIVSNKPYFYYPAVQANAKHVVDVSDLDAIPRVDILYAYEDMQTDSIYSAVQNGAKGVVIAGQGAGGVSTDFASAINDIVAKHNIPVVLSHRTVNGEVPTAAITAKNAQTPIASGMFNPQQSRILLGLLLAEKKGLVEIREVFSKATVA

> KGO77393.1 L-asparaginase *Penicillium italicum*

MGLTLQTLAFSALAITSYASPLIYPRAANTSCTNSNGLTFNHFNGSLPNVTILATGGTIAGTSDDKTATAGYKSGALGINTILSEIPDIFNVANIAAVQAHNVNSGDITSALLLNLTHTLQRQVCDDPTMSGAVITHGTDTLEESAFFIDATVNCGKPIVFVGSMRPSTALSADGAMNLLHGVTVAADKNSGDRGALVVLNDRIVSALFATKTNANTIDTFKAYEQGSLGFIVSNKPYFYYPAVQANAKHVADVSNVDAVPRVDILYAYEDMQVDSIYSAVNNGAKGIVIAGEGAGGVTTDFASAINDIGAKHNIPIVLSHRTVNGEVPTADFTGENSGTKIASGMFNPQQSRVLLGLLLAEKKGLEEIREVFLKAAVA

> MW291568 L-asparaginase *Penicillium sizovae*

MVSIKSFLVALATFACQSSASPLLYGRGTNGTGFVFTNANGLNFTQMNHTLPNITIFATGGTIAGSDSSSTATTGYTSGAVGVRALIDAVPSMLDIANVAGVQTANVGSEDITSDILISLSKQINKFVCDDPTMAGAVVTHGTDTLEETAFFLDATINCGKPVIIVGAMRPSTAISADGPFNLLESVTVAASPKAKNRGAMIVMNDRIASAYYTTKTNANTMDTFKAMEMGYLGEMISNTPFFFYPPVQPTGKKDFNIANVTEIPRVDILFSYEDMHNDTLYNAIESGAKGIVIAGAGAGGVTTSFNYAIEDAINRLGIPIIQSMRTVNGEVPLSDVESTSATHIASGYLNPQKSRILLGLLLAKSSNITEIASTFSLNTNA

> OQE28485.1 L-asparaginase *Penicillium steckii*

MVPIKSFLVALATLAWQSSASPLLYSRGANGTGFVFTNANGLNFTQMNHTLPNITIFATGGTIAGSDSSSTATTGYTSGAVGVRALIDAVPSMLDIANVAGVQTANVGSEDITSDILISLSKQINKLVCDDSTMAGAVVTHGTDTLEETAFFLDATVNCGKPVIIVGAMRPSTAISADGPFNLLESVTVAASTKAKNRGAMIVMNDRIASAYYTTKTNANTMDTFKAMEMGFLGEMISNTPFFFYPPVQPTGKKDFDISNVKEIPRVDILFSYEDMHNDTLYNAVESGAKGIVIAGAGAGGVTTSFNYAMEDVINRLGIPIIQSMRTVNGEVPLSDVESNSATHIASGYLNPQKSRILLGLLLAKSSNITEIASTFALNTNA

> PYI32151.1 L-asparaginase *Aspergillus idologenus*

MAIKSVVLSALATLASASPLLHARDSNSTTYVFTNSNGLNFTQMNASLPNVTIFATGGTIAGSDSSSTATTGYTSGAVGVLTLIDAVPSMLDVANVAGVQVANVGSEDITSDILISLSKDITRMVCDDPTMAGAVITHGTDTLEESAFFMDATVNCGKPVVIVGAMRPSTAISADGPFNLLEAVTVAASPKARDRGAMVVMNDRIASAYYVTKTNANTMDTFKAMEMGFLGEMISNNPFFFYPPVTPTGKVPFDIANVTEIPRVDILFSYEDMHNDTLYNAVESGAKGIVIAGAGAGGVTTSFNAAIEDVINRLEIPVVQSMRTVNGEVPLSDVSSDTATHIASGYLNPQKSRVLLGLLLAEGKNLTEIADVFALGTNA

> XP_025570260.1 L-asparaginase *Aspergillus ibericus*

MTIKPLLLSALATLGSASPLLYPRATNETYVFTNANGLNFTQMNTSLPNVTIFATGGTIAGSDSSSTATTGYTSGAVGVLTLIDAVPSMLDVANVAGVQVANVGSEDITSDILISMSKEINRVVCDDPTMAGAVITHGTDTLEETAFFMDATINCGKPVVIVGAMRPSTAISADGPFNLLEAVTVAASPKARDRGAMVVMNDRIASAYYVTKTNANTMDTFKAMEMGYLGEMISNTPFFFYPPVKPTGKVAFDITNVTEIPRVDILFSYEDMHNDTLYNAIESGAEGIVIAGAGAGGVTTSFNYAIEDVINRLDIPVVQSMRTVNGEVPLSDVSSDTATHIASGYLNPQKSRILLGLLLAEGKNLTEIADVFALGTDA

> PYI04731.1 L-asparaginase *Aspergillus sclerotiicarbonarius*

MTFKPLLLSALATLGSASPLLHPRATNETYVFTNANGLNFTQMNTSLPNVTIFATGGTIAGSDSSSTATTGYTSGAVGVLTLIDAVPTMLDVANVAGVQVANVGSEDITSDILISMSKEINRVVCDDPTMAGAVITHGTDTLEETAFFMDATINCGKPVVIVGAMRPSTAISADGPFNLLEAVTVAASPKARDRGAMVVMNDRIASAYYVTKTNANTMDTFKAMEMGYLGEMISDTPFFFYPPVKPTGKVAFDIANVTEIPRVDILFSYEDMHNDTLYNAIESGAEGIVIAGAGAGGVTTSFNYAIEDAINRLEIPVVQSMRTVNGEVPLSDISSDTATHIASGYLNPQKSRILLGLLLAEGKNITEIADVFALGTDA

> XP 001389884.1 L-asparaginase *Aspergillus niger*

MPLKPILLSALASLASASPLLYSRTTNETFVFTNANGLNFTQMNTTLPNVTIFATGGTIAGSDSSSTATTGYTSGAVGVLSLIDAVPSMLDVANVAGVQVANVGSEDITSDILISMSKKLNRVVCEDPTMAGAVITHGTDTLEETAFFLDATVNCGKPIVIVGAMRPSTAISADGPFNLLEAVTVAASTSARDRGAMVVMNDRIASAYYVTKTNANTMDTFKAMEMGYLGEMISNTPFFFYPPVKPTGKVAFDITNVTEIPRVDILFSYEDMHNDTLYNAISSGAQGIVIAGAGAGGVTTSFNEAIEDVINRLEIPVVQSMRTVNGEVPLSDVSSDTATHIASGYLNPQKSRILLGLLLSQGKNITEIADVFALGTDA

> 3ECA L-asparaginase *Escherichia coli*

LPNITILATGGTIAGGGDSATKSNYTAGKVGVENLVNAVPQLKDIANVKGEQVVNIGSQDMNDDVWLTLAKKINTDCDKTDGFVITHGTDTMEETAYFLDLTVKCDKPVVMVGAMRPSTSMSADGPFNLYNAVVTAADKASANRGVLVVMNDTVLDGRDVTKTNTTDVATFKSVNYGPLGYIHNGKIDYQRTPARKHTSDTPFDVSKLNELPKVGIVYNYANASDLPAKALVDAGYDGIVSAGVGNGNLYKTVFDTLATAAKNGTAVVRSSRVPTGATTQDAEVDDAKYGFVASGTLNPQKARVLLQLALTQTKDPQQIQQIFNQY

> 2JK0 L-asparaginase *Erwinia carotovora* (*Dickeya chrysanthemi*)

NLPNIVILATGGTIAGSAAANTQTTGYKAGALGVETLIQAVPELKTLANIKGEQVASIGSENMTSDVLLTLSKRVNELLARSDVDGVVITHGTDTLDESPYFLNLTVKSDKPVVFVAAMRPATAISADGPMNLYGAVKVAADKNSRGRGVLVVLNDRIGSARFISKTNASTLDTFKAPEEGYLGVIIGDKIYYQTRLDKVHTTRSVFDVTNVDKLPAVDIIYGYQDDPEYMYDASIKHGVKGIVYAGMGAGSVSKRGDAGIRKAESKGIVVVRSSRTGSGIVPPDAGQPGLVADSLSPAKSRILLMLALTKTTNPAVIQDYFHAY
