# Supplementary material for: A Structural In Silico Analysis of the Immunogenicity of L-Asparaginase from Penicillium cerradense
Source: Int J Mol Sci. 2024 Apr 27;25(9):4788. doi: 10.3390/ijms25094788 (PMC11084778; doi:10.3390/ijms25094788)
Supplement: Supplementary file 1 [file ijms-25-04788-s001.zip › Supplementary/Supplementary Table S2- Epitopos alergenicos HLA07 01.docx]

**Supplementary Table S2**

T-cell epitopes predicted as allergenic for the HLA-DRB1*07:01 allele.

| *Escherichia coli* | *Dickeia chrysanthemi* | *Penicillium cerradense* | *Penicillium chrysogenum* | *Penicillium digitatum* | *Penicillium griseofulvum* | *Penicillium italicum* | *Penicillium sizovae* | *Penicillium steckii* |
| --- | --- | --- | --- | --- | --- | --- | --- | --- |
| LPNITILATGGTIAG  PNITILATGGTIAGG  QDMNDDVWLTLAKKI  DMNDDVWLTLAKKIN  MNDDVWLTLAKKINT  DDVWLTLAKKINTDC  DVWLTLAKKINTDCD  CDKTDGFVITHGTDT  DKTDGFVITHGTDTM  TDGFVITHGTDTMEE  DGFVITHGTDTMEET  VMVGAMRPSTSMSAD  MVGAMRPSTSMSADG  VGAMRPSTSMSADGP  GAMRPSTSMSADGPF  SADGPFNLYNAVVTA  GPFNLYNAVVTAADK  PFNLYNAVVTAADKA  FNLYNAVVTAADKAS  NLYNAVVTAADKASA  LDGRDVTKTNTTDVA  DGRDVTKTNTTDVAT  GRDVTKTNTTDVATF  TNTTDVATFKSVNYG  NTTDVATFKSVNYGP  GPLGYIHNGKIDYQR  HNGKIDYQRTPARKH  NGKIDYQRTPARKHT  GKIDYQRTPARKHTS  KIDYQRTPARKHTSD  DTPFDVSKLNELPKV  TPFDVSKLNELPKVG  ALVDAGYDGIVSAGV  LVDAGYDGIVSAGVG  VDAGYDGIVSAGVGN  YKTVFDTLATAAKNG  AAKNGTAVVRSSRVP  AKNGTAVVRSSRVPT  KNGTAVVRSSRVPTG  EVDDAKYGFVASGTL  VDDAKYGFVASGTLN | NLPNIVILATGGTIA  LPNIVILATGGTIAG  PNIVILATGGTIAGS  PELKTLANIKGEQVA  ELKTLANIKGEQVAS  KGEQVASIGSENMTS  GEQVASIGSENMTSD  DVDGVVITHGTDTLD  VDGVVITHGTDTLDE  DGVVITHGTDTLDES  DESPYFLNLTVKSDK  ESPYFLNLTVKSDKP  SDKPVVFVAAMRPAT  DKPVVFVAAMRPATA  VFVAAMRPATAISAD  FVAAMRPATAISADG  AAMRPATAISADGPM  SADGPMNLYGAVKVA  ADGPMNLYGAVKVAA  DGPMNLYGAVKVAAD  GPMNLYGAVKVAADK  PMNLYGAVKVAADKN  MNLYGAVKVAADKNS  NLYGAVKVAADKNSR  IGSARFISKTNASTL  IGDKIYYQTRLDKVH  GDKIYYQTRLDKVHT  DKIYYQTRLDKVHTT  TRSVFDVTNVDKLPA  RSVFDVTNVDKLPAV  SVFDVTNVDKLPAVD  QDDPEYMYDASIKHG  DDPEYMYDASIKHGV  DPEYMYDASIKHGVK  KGIVYAGMGAGSVSK  GIVYAGMGAGSVSKR  KRGDAGIRKAESKGI  GDAGIRKAESKGIVV  KAESKGIVVVRSSRT  AESKGIVVVRSSRTG  ESKGIVVVRSSRTGS  SKGIVVVRSSRTGSG  KGIVVVRSSRTGSGI  GLVADSLSPAKSRIL  LVADSLSPAKSRILL  VADSLSPAKSRILLM  LLMLALTKTTNPAVI  MLALTKTTNPAVIQD  LALTKTTNPAVIQDY | QSSSTKYVYTNSNGL  SSSTKYVYTNSNGLN  TKYVYTNSNGLNFTQ  KYVYTNSNGLNFTQM  PNITIFGTGGTIAGS  NITIFGTGGTIAGSG  LNVSNIAGVQVANVG  NVSNIAGVQVANVGS  VSNIAGVQVANVGSE  SNIAGVQVANVGSED  IAGVQVANVGSEDIT  AGVQVANVGSEDITS  GVQVANVGSEDITSD  VIVGAMRPSTATSAD  IVGAMRPSTATSADG  ADGPFNLLEAVTVAA  DGPFNLLEAVTVAAS  GPFNLLEAVTVAASP  LEAVTVAASPKAVNR  DRIASAYYVTKTNAN  IASAYYVTKTNANTM  ASAYYVTKTNANTMD  SAYYVTKTNANTMDT  AYYVTKTNANTMDTF  PTGKVEFDITATKEI  TGKVEFDITATKEIP  KVEFDITATKEIPRV  EFDITATKEIPRVDI  AGAGGVSTSFNHAIE  GAGGVSTSFNHAIED  AGGVSTSFNHAIEDV  GGVSTSFNHAIEDVI  GVSTSFNHAIEDVIN  EVPLSDVNSTSAIHI  VPLSDVNSTSAIHIA  PLSDVNSTSAIHIAS  LSDVNSTSAIHIASG  SDVNSTSAIHIASGY  NSTSAIHIASGYLNP  RILLGLLLSEARTLT  LLGLLLSEARTLTDI | PEIFNVANIAAVQAH  SYASPLIHSRATNTS  YASPLIHSRATNTSY  ASPLIHSRATNTSYT  SRATNTSYTNSNGLT  RATNTSYTNSNGLTF  ATNTSYTNSNGLTFN  TNTSYTNSNGLTFNH  TSYTNSNGLTFNHFN  LPNVTILATGGTIAG  PNVTILATGGTIAGT  NVTILATGGTIAGTS  KIPEIFNVANIAAVQ  IPEIFNVANIAAVQA  IFNVANIAAVQAHNV  NVANIAAVQAHNVNS  VANIAAVQAHNVNSG  ANIAAVQAHNVNSGD  NCGKPIVFVGSMRPA  GKPIVFVGSMRPATA  VSALFATKTHANTMD  SALFATKTHANTMDT  SNKPYFYYPAVQANV  NKPYFYYPAVQANVK  KPYFYYPAVQANVKH  PYFYYPAVQANVKHV  ATDFGSAINDIVKKH  AINDIVKKHNIPVVL  INDIVKKHNIPVVLS | SALVTTSYASPLIYP  PRAVNTSYTNSNGLT  RAVNTSYTNSNGLTF  AVNTSYTNSNGLTFS  VNTSYTNSNGLTFSH  NTSYTNSNGLTFSHF  TSYTNSNGLTFSHFN  LPNVTILATGGTIAG  PNVTILATGGTIAGT  NVTILATGGTIAGTS  SGIPDIFKIANIAAI  GIPDIFKIANIAAIQ  IPDIFKIANIAAIQA  PDIFKIANIAAIQAH  DIFKIANIAAIQAHN  IFKIANIAAIQAHNV  IANIAAIQAHNVNSG  IVFVGSMRPSTAISA  VFVGSMRPSTAISAD  FVGSMRPSTAISADG  SALFATKTSANTVDT  ALFATKTSANTVDTF  SNKPYFYYPAVQANA  NKPYFYYPAVQANAK  KPYFYYPAVQANAKH  PYFYYPAVQANAKHV  FYYPAVQANAKHVAD  SAINDIGVKHSIPVV  INDIGVKHSIPVVLS  NDIGVKHSIPVVLSH  IASGMFNPQQSRILL  ASGMFNPQQSRILLG  SGMFNPQQSRILLGL | SYASPLIHSRATNTS  YASPLIHSRATNTSY  ASPLIHSRATNTSYT  SRATNTSYTNSNGLT  RATNTSYTNSNGLTF  TANTSYTNSNGLTFN  ANTSYTNSNGLTFNH  TSYTNSNGLTFNHFN  LPNVTILATGGTIAG  PNVTILATGGTIAGT  NVTILATGGTIAGTS  KIPEIFNVANIAAVQ  IPEIFNVANIAAVQA  PDIFNVANIAAIQAN  PEIFNVANIAAVQAH  IFNVANIAAIQANNV  NVANIAAIQANNVNS  VANIAAIQANNVNSG  ANIAAIQANNVNSGD  NCGKPIVFVGSMRPA  GKPIVFVGSMRPATA  VFVGSMRPSTAISAD  FVGSMRPSTAISADG  VSAFFATKTHANTMD  VSALFATKTHANTMD  SALFATKTHANTMDT  SNKPYFYYPAVQANA  NKPYFYYPAVQANAK  KPYFYYPAVQANAKH  PYFYYPAVQANVKHV  ATDFGSAINDIVKKH  AINDIVKKHNIPVVL  INDIVKKHNIPVVLS | ALAITSYASPLIYPR  LPNVTILATGGTIAG  PNVTILATGGTIAGT  NVTILATGGTIAGTS  EIPDIFNVANIAAVQ  PDIFNVANIAAVQAH  IFNVANIAAVQAHNV  NVANIAAVQAHNVNS  VANIAAVQAHNVNSG  ANIAAVQAHNVNSGD  IVFVGSMRPSTALSA  VFVGSMRPSTALSAD  RIVSALFATKTNANT  IVSALFATKTNANTI  SALFATKTNANTIDT  ALFATKTNANTIDTF  SNKPYFYYPAVQANA  NKPYFYYPAVQANAK  KPYFYYPAVQANAKH  PYFYYPAVQANAKHV  FYYPAVQANAKHVAD  QVDSIYSAVNNGAKG  VDSIYSAVNNGAKGI  IASGMFNPQQSRVLL  ASGMFNPQQSRVLLG  SGMFNPQQSRVLLGL | GTNGTGFVFTNANGL  TNGTGFVFTNANGLN  LPNITIFATGGTIAG  PNITIFATGGTIAGS  NITIFATGGTIAGSD  IANVAGVQTANVGSE  IIVGAMRPSTAISAD  VIIVGAMRPSTAISA  IVGAMRPSTAISADG  GAMRPSTAISADGPF  ADGPFNLLESVTVAA  DGPFNLLESVTVAAS  LLESVTVAASPKAKN  LESVTVAASPKAKNR  DRIASAYYTTKTNAN  ASAYYTTKTNANTMD  KKDFNIANVTEIPRV  AKGIVIAGAGAGGVT  AGAGGVTTSFNYAIE  GAGGVTTSFNYAIED  AGGVTTSFNYAIEDA  GGVTTSFNYAIEDAI  GVTTSFNYAIEDAIN  PLSDVESTSATHIAS  LSDVESTSATHIASG  SDVESTSATHIASGY  DVESTSATHIASGYL  LLGLLLAKSSNITEI  SNITEIASTFSLNTN  NITEIASTFSLNTNA | ATLAWQSSASPLLYS  TLAWQSSASPLLYSR  LAWQSSASPLLYSRG  SASPLLYSRGANGTG  LPNITIFATGGTIAG  PNITIFATGGTIAGS  NITIFATGGTIAGSD  IANVAGVQTANVGSE  VIIVGAMRPSTAISA  IIVGAMRPSTAISAD  IVGAMRPSTAISADG  GAMRPSTAISADGPF  ADGPFNLLESVTVAA  DGPFNLLESVTVAAS  LLESVTVAASTKAKN  LESVTVAASTKAKNR  ESVTVAASTKAKNRG  DRIASAYYTTKTNAN  ASAYYTTKTNANTMD  GKKDFDISNVKEIPR  GVTTSFNYAMEDVIN  PLSDVESNSATHIAS  LSDVESNSATHIASG  LLGLLLAKSSNITEI  LGLLLAKSSNITEIA  SNITEIASTFALNTN  NITEIASTFALNTNA |

| *Aspergillus ibericus* | *Aspergillus idologenus* | *Aspergillus niger* | *Aspergillus sclerotiicarbonarius* |  |  |  |  |  |
| --- | --- | --- | --- | --- | --- | --- | --- | --- |
| VIVGAMRPSTAISAD  GVQVANVGSEDITSD  NVTIFATGGTIAGSD  GAMRPSTAISADGPF  AGVQVANVGSEDITS  AGGVTTSFNYAIEDV  GAGGVTTSFNYAIED  EDITSDILISMSKEI  PNVTIFATGGTIAGS  DRIASAYYVTKTNAN  VAGVQVANVGSEDIT  GPFNLLEAVTVAASP  LPNVTIFATGGTIAG  DTLYNAIESGAEGIV  NDTLYNAIESGAEGI  VANVAGVQVANVGSE  GVTTSFNYAIEDVIN  LEAVTVAASPKARDR  AGAGGVTTSFNYAIE  VVIVGAMRPSTAISA  HNDTLYNAIESGAEG  DVANVAGVQVANVGS  LLEAVTVAASPKARD  LDVANVAGVQVANVG  GGVTTSFNYAIEDVI  PSMLDVANVAGVQVA  ATNETYVFTNANGLN  KVAFDITNVTEIPRV  GKVAFDITNVTEIPR  ASAYYVTKTNANTMD  DITSDILISMSKEIN  SAYYVTKTNANTMDT  IVGAMRPSTAISADG  DGPFNLLEAVTVAAS  IASAYYVTKTNANTM  AYYVTKTNANTMDTF  ADGPFNLLEAVTVAA  VAFDITNVTEIPRVD | DSNSTTYVFTNSNGL  NSTTYVFTNSNGLNF  STTYVFTNSNGLNFT  TTYVFTNSNGLNFTQ  TYVFTNSNGLNFTQM  LPNVTIFATGGTIAG  PNVTIFATGGTIAGS  NVTIFATGGTIAGSD  PSMLDVANVAGVQVA  LDVANVAGVQVANVG  DVANVAGVQVANVGS  VANVAGVQVANVGSE  VAGVQVANVGSEDIT  AGVQVANVGSEDITS  GVQVANVGSEDITSD  EDITSDILISLSKDI  VVIVGAMRPSTAISA  VIVGAMRPSTAISAD  IVGAMRPSTAISADG  GAMRPSTAISADGPF  ADGPFNLLEAVTVAA  DGPFNLLEAVTVAAS  GPFNLLEAVTVAASP  LLEAVTVAASPKARD  LEAVTVAASPKARDR  DRIASAYYVTKTNAN  IASAYYVTKTNANTM  ASAYYVTKTNANTMD  SAYYVTKTNANTMDT  AYYVTKTNANTMDTF  PTGKVPFDIANVTEI  KVPFDIANVTEIPRV  VPFDIANVTEIPRVD  AGAGGVTTSFNAAIE  GAGGVTTSFNAAIED  AGGVTTSFNAAIEDV | TTNETFVFTNANGLN  TNETFVFTNANGLNF  RTTNETFVFTNANGL  ETFVFTNANGLNFTQ  LPNVTIFATGGTIAG  PNVTIFATGGTIAGS  NVTIFATGGTIAGSD  PSMLDVANVAGVQVA  LDVANVAGVQVANVG  DVANVAGVQVANVGS  VANVAGVQVANVGSE  VAGVQVANVGSEDIT  AGVQVANVGSEDITS  GVQVANVGSEDITSD  CGKPIVIVGAMRPST  IVIVGAMRPSTAISA  VIVGAMRPSTAISAD  IVGAMRPSTAISADG  GAMRPSTAISADGPF  ADGPFNLLEAVTVAA  DGPFNLLEAVTVAAS  GPFNLLEAVTVAAST  LEAVTVAASTSARDR  EAVTVAASTSARDRG  AVTVAASTSARDRGA  VTVAASTSARDRGAM  DRIASAYYVTKTNAN  IASAYYVTKTNANTM  ASAYYVTKTNANTMD  SAYYVTKTNANTMDT  AYYVTKTNANTMDTF  KVAFDITNVTEIPRV  VAFDITNVTEIPRVD  HNDTLYNAISSGAQG  NDTLYNAISSGAQGI  DTLYNAISSGAQGIV  AGAGGVTTSFNEAIE  GAGGVTTSFNEAIED  AGGVTTSFNEAIEDV | ATNETYVFTNANGLN  LPNVTIFATGGTIAG  PNVTIFATGGTIAGS  NVTIFATGGTIAGSD  PTMLDVANVAGVQVA  TMLDVANVAGVQVAN  LDVANVAGVQVANVG  DVANVAGVQVANVGS  VANVAGVQVANVGSE  VAGVQVANVGSEDIT  AGVQVANVGSEDITS  GVQVANVGSEDITSD  EDITSDILISMSKEI  DITSDILISMSKEIN  VVIVGAMRPSTAISA  VIVGAMRPSTAISAD  IVGAMRPSTAISADG  GAMRPSTAISADGPF  ADGPFNLLEAVTVAA  DGPFNLLEAVTVAAS  GPFNLLEAVTVAASP  LLEAVTVAASPKARD  LEAVTVAASPKARDR  DRIASAYYVTKTNAN  IASAYYVTKTNANTM  ASAYYVTKTNANTMD  SAYYVTKTNANTMDT  AYYVTKTNANTMDTF  PTGKVAFDIANVTEI  KVAFDIANVTEIPRV  VAFDIANVTEIPRVD  HNDTLYNAIESGAEG  NDTLYNAIESGAEGI  DTLYNAIESGAEGIV  AGAGGVTTSFNYAIE  GAGGVTTSFNYAIED  AGGVTTSFNYAIEDA  GGVTTSFNYAIEDAI  GVTTSFNYAIEDAIN  LSDISSDTATHIASG |  |  |  |  |  |
